# Neurovascular Issues in Antiphospholipid Syndrome: Arterial Vasculopathy from Small to Large Vessels in a Neuroradiological Perspective

**DOI:** 10.3390/jcm13133667

**Published:** 2024-06-24

**Authors:** Marialuisa Zedde, Ilaria Grisendi, Federica Assenza, Manuela Napoli, Claudio Moratti, Bonacini Lara, Giovanna Di Cecco, Serena D’Aniello, Claudio Pavone, Francesca Romana Pezzella, Paolo Candelaresi, Vincenzo Andreone, Franco Valzania, Rosario Pascarella

**Affiliations:** 1 Neurology Unit, Stroke Unit, Azienda Unità Sanitaria Locale-IRCCS di Reggio Emilia, Viale Risorgimento 80, 42123 Reggio Emilia, Italy; grisendi.ilaria@ausl.re.it (I.G.); assenza.federica@ausl.re.it (F.A.); valzania.franco@ausl.re.it (F.V.); 2 Neuroradiology Unit, Azienda Unità Sanitaria Locale-IRCCS di Reggio Emilia, Viale Risorgimento 80, 42123 Reggio Emilia, Italy; napoli.manuela@ausl.re.it (M.N.); moratti.claudio@ausl.re.it (C.M.); bonacini.lara@ausl.re.it (B.L.); dicecco.giovanna@ausl.re.it (G.D.C.); daniello.serena@ausl.re.it (S.D.); pavone.claudio@ausl.re.it (C.P.); pascarella.rosario@ausl.re.it (R.P.); 3 Stroke Unit, Department of Neuroscience, San Camillo Forlanini Hospital, 00152 Rome, Italy; frpezzella@gmail.com; 4 Neurology and Stroke Unit, AORN Antonio Cardarelli, 80131 Naples, Italy; paolo.candelaresi@aocardarelli.it (P.C.); andreone2@gmail.com (V.A.)

**Keywords:** antiphospholipid syndrome, APS, large vessels, SVD, MRI, neuroradiology, moyamoya, proliferative vasculopathy, intracranial occlusion, vasculitis

## Abstract

Antiphospholipid syndrome (APS) is an autoimmune prothrombotic condition characterized by venous thromboembolism, arterial thrombosis, and pregnancy morbidity. Among neurological manifestations, arterial thrombosis is only one of the possible associated clinical and neuroradiological features. The aim of this review is to address from a neurovascular point of view the multifaceted range of the arterial side of APS. A modern neurovascular approach was proposed, dividing the CNS involvement on the basis of the size of affected arteries, from large to small arteries, and corresponding clinical and neuroradiological issues. Both large-vessel and small-vessel involvement in APS were detailed, highlighting the limitations of the available literature in the attempt to derive some pathomechanisms. APS is a complex disease, and its neurological involvement appears multifaceted and not yet fully characterized, within and outside the diagnostic criteria. The involvement of intracranial large and small vessels appears poorly characterized, and the overlapping with the previously proposed inflammatory manifestations is consistent.

## 1. Introduction

Antiphospholipid syndrome (APS) is defined as it was in the 1980s, referring to a condition of autoantibody-induced thrombophilia [1,2]. The main clinical features of APS are included in the original description of this autoimmune prothrombotic condition: venous thromboembolism, arterial thrombosis, and pregnancy morbidity. The diagnosis is strongly supported by the laboratory evidence of elevated levels of antiphospholipid antibodies (aPLs): lupus anticoagulant (LAC) (clot-based assays), anticardiolipin (aCL), and anti–β2-glycoprotein 1 (β2-GP1) antibodies (immunoglobulin G or immunoglobulin M) (enzyme-linked immunosorbent assays). The name of the disease depends on the targets of autoantibodies; in fact, this class of antibodies targets antiphospholipid-bound proteins. It is of paramount importance to not base the diagnosis only on the laboratory side, there being reported a high prevalence of aPLs in cohorts of normal subjects. In fact, aPLs are found in 1–5% of the general population, with a higher prevalence observed in older individuals [3]. However, it is important to note that only a subset of these individuals actually develops APS [3]. The estimated prevalence of APS is approximately 40–50 per 100,000 individuals, with an annual incidence of 5 cases per 100,000 individuals [4]. In a random sample of 552 healthy blood donors, the prevalence of aPLs was found to be 6.5% and 9.4% for aCL immunoglobulin G and immunoglobulin M antibodies, respectively, but none of those normal subjects with positive aPLs developed thrombotic events at 1-year follow-up [5]. In a subsequent investigation, which involved a follow-up study with a mean duration of 9.1 ± 7.5 years (range: 3–41 years), it was discovered that asymptomatic carriers of aPLs face an increased risk of future thrombotic events, particularly when they test positive for double or triple aPL positivity. Notably, individuals with an underlying autoimmune disease were exclusively affected by thrombotic events during the follow-up period. Conversely, single positivity for aPLs did not appear to elevate the risk of thrombosis [6].

Thrombosis is one of the main clinical features of APS, occurring in 166/1000 (16.6%) patients during the first 5-year study period and in 115/1000 (14.4%) during the second 5-year period in an observational multinational European study [7]. Moreover, the presence of vascular risk factors (hypertension and hypertriglyceridemia) increased the risk of arterial thrombosis in APS patients with positive aCL [8]. Thrombosis in APS follows the “20% rule”. This rule suggests that APS accounts for up to 20% of unprovoked deep vein thrombosis (DVT), 20% of strokes in young adults (under 50 years), and up to 20% of women with recurrent fetal loss. However, more recent updates have refined these figures to approximately 20% of DVT cases, 20–30% of strokes in young adults (under 50 years), and 10–15% of women experiencing recurrent fetal loss [9,10].

Many other neurovascular manifestations have been described, but they are essentially neglected and poorly systematized in the available literature. The aim of this review is to systematically describe arterial cerebrovascular involvement in patients with APS, adopting a distinct neurovascular approach both clinically and neuroradiologically.

## 2. Updated Classification Criteria and Their Implication on Neurovascular Issues

After its first definition, APS was characterized as a systemic autoimmune disease with arterial, venous, or microvascular thrombosis, pregnancy morbidity, or nonthrombotic manifestations in patients with persistent aPL. The first classification of APS was proposed in the Sapporo criteria published in 1999 [11] and revised in 2006 [12]. According to the revised Sapporo criteria, for the diagnosis of APS, both the following issues are needed:

(1) clinical features: thrombosis or pregnancy morbidity;
(2) laboratory tests (LAC, IgG/IgM aCL, and/or IgG/IgM anti-β2GPI) with at least two aPL tests performed at least 12 weeks apart.

**Figure 1:**
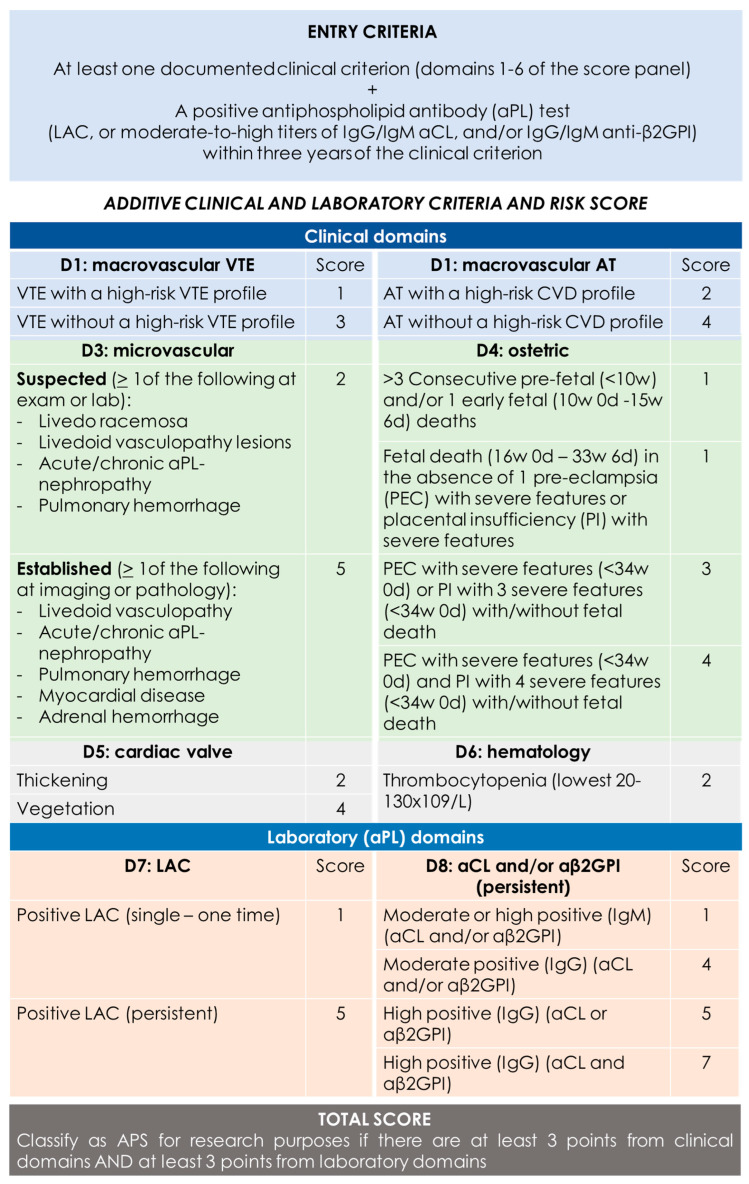
2023 ACR/EULAR APS classification criteria [14]. “Persistent” aPL test results (at least 12 weeks apart) should be scored based on two consecutive positive LAC, two consecutive highest aCL, and/or two consecutive highest aβ2GPI results (two consecutive results with one moderate positive and one high positive aCL/aβ2GPI should be marked as “moderate positive” if there are no additional consecutive high results available). CVD = cardiovascular disease; VTE = venous thromboembolism; AT = arterial thrombosis.

A definite diagnosis of APS requires the presence of at least one clinical and one laboratory criterion. Clinical criteria may include objectively confirmed venous, arterial, or small-vessel thrombosis or pregnancy morbidity attributable to placental insufficiency, including pregnancy loss or premature birth. Laboratory criteria encompass persistently positive test results for at least one of these three aPLs measures on two or more occasions 12 weeks apart. The 12-week testing interval is particularly important, given that some infections and medications can cause transient aPL-positive testing [13]. These criteria carry several limitations, such as the exclusion of some clinical manifestations, and overlook conventional vascular risk factors or the different risk of thrombosis associated with a different antibody profile. Therefore, in 2023, new diagnostic criteria were proposed, which are summarized in Figure 1. [14].

Among clinical domains, neurovascular issues are included in domain 1 macrovascular (VTE as cerebral venous thrombosis—CVT) and in domain 2 macrovascular AT as “stroke based on international definitions” [15,16]. Thus, stroke etiology is defined according to the TOAST and ASCOD classifications. The microvascular domain (3) does not include any neurovascular manifestations, and there are no items referring to extracranial and intracranial vessels neither large nor small. Another crucial issue is the definition of high cardiovascular risk, used for identifying an alternative cause for arterial thrombosis. In the updated classification, the following scheme is proposed (Table 1):

One notable advancement in the updated APS classification involves explicitly considering potential causes beyond APS for both arterial and venous thrombosis. This includes defining a high-risk VTE or high-risk CVD profile to prevent overestimating the contribution of aPL to thrombosis. Moreover, the classification now requires scoring “persistent” aPL based on two consecutive results. Additionally, aCL/anti-β2GPI positivity is categorized as “moderate” if it falls within 40–79 ELISA units and “high” if it is ≥80 ELISA units.

However, the issue of VTE or AT alone, particularly in patients with high-risk VTE or CVD profiles and laboratory criteria scoring ≥3, is proposed as a priority for future research.

These criteria were originally proposed in order to standardize the definition of the disease and its subtypes for clinical studies, and they were not validated as diagnostic criteria for APS in clinical practice. This is a relevant limitation, but the updated classification might help to rationalize the pathophysiology of APS manifestations and organ involvement.

## 3. Neurological Manifestations in APS

Cerebrovascular disease emerges as the primary cause of neurological symptoms in APS patients, playing a central role in APS classification due to its thromboembolic nature. This includes acute ischemic stroke and transient ischemic attacks (TIAs), both recognized consequences of APS. Furthermore, cerebral venous thrombosis (CVT) becomes evident, along with less common conditions such as Sneddon’s syndrome and reversible cerebral vasoconstriction syndrome. The cerebral circulation stands out as the most frequent site of arterial thrombosis in APS. Ischemic stroke or TIA serve as the initial presentation in almost 30% of adults with APS [17]. However, recent evidence suggests that APS-associated neurologic dysfunction extends beyond classical thromboembolic events and involves immune-mediated vascular, inflammatory, and direct neuronal effects [18]. Increased permeability of the blood–brain barrier (BBB) can result from both small/microvessel thrombosis, leading to subsequent ischemia, and the direct effects of antiphospholipid antibodies [19]. These antibodies have been shown to trigger leukocyte adhesion and complement activation, further disrupting the barrier and resulting in neurotoxicity from cytokines and antibodies [19].

Cerebrovascular diseases stand out as the primary concern among CNS manifestations of APS, encompassing both venous and arterial macrovascular clinical domains. This includes acute ischemic stroke and TIA, well-recognized risks associated with APS. Additionally, CVT and other less common disorders like Sneddon’s syndrome and reversible cerebral vasoconstriction syndrome are observed. In the largest series published to date, involving 1000 patients with a 10-year follow-up, stroke occurred in 198 out of 1000 patients (19.8%) initially, with 53 out of 1000 patients (5.3%) experiencing incident stroke by the end of the follow-up period. Interestingly, strokes were slightly more prevalent in SLE-associated APS compared to primary APS (*p* = 0.036). Notably, stroke accounted for 11 out of 93 patient deaths (11.8%) during the follow-up. Arterial thrombosis primarily affects the cerebral circulation, with ischemic stroke or transient ischemic attack (TIA) being the initial presentation in nearly 30% of adults with APS [17].

Conversely, the prevalence of positive aPLs among stroke patients fluctuates between 7% and 15% [20], displaying an apparent correlation between aPL positivity and age in stroke cases: aPL-positive stroke patients tend to be younger on average than the general population [21,22,23]. APS is implicated in a significant proportion of acute ischemic strokes in younger individuals [24], with aPL presence correlating with over a 5-fold surge in cerebrovascular thrombotic events (odds ratio [OR] of 5.48, 95% confidence interval [CI]: 4.42 to 6.79) among stroke patients under 50 years of age (median age 37 years) [25]. However, in older patients, aPL positivity might carry less weight as a risk factor due to the presence of competing cardiovascular risk factors, variations in the effects of different aPLs, and potential biases stemming from study designs that often exclude patients with cardioembolic strokes [26,27]. The etiology of stroke in APS is predominantly attributed to either thrombotic or cardioembolic mechanisms. Nonetheless, intracranial steno-occlusions are found in half of APS patients with stroke [22], hinting at a concurrent vasculopathic process in some instances [28,29,30]. Cardioembolic pathways encompass left-sided cardiac valve irregularities (characterized by irregular thickening due to immune complex deposition, vegetations, valve dysfunction) and, in rare cases, intracardiac thrombi [31,32,33]. Additionally, small-vessel cerebrovascular disease is frequently documented, contributing to lacunar and subcortical strokes [34].

CVT emerges as a rare complication of APS, boasting a documented prevalence of 0.7% [17]. Conversely, APS plays a notable role in a substantial fraction of CVT occurrences, accounting for 6–17% of cases in cohort studies [35]. Additionally, aCL positivity is detected in 7–22% of patients with CVT [36,37], with CVT sometimes serving as the initial indication of APS [38]. Treatment typically adheres to general CVT guidelines [39], often involving long-term anticoagulation. However, more recent evidence supports the concept that APS-associated neurologic dysfunction extends beyond the classical thromboembolic events and is also related to immune-mediated vascular, inflammatory, and direct neuronal effects [18].

Sneddon’s syndrome presents as a gradually advancing noninflammatory thrombotic vasculopathy distinguished by livedo racemosa and recurrent cerebrovascular incidents, encompassing both ischemic and hemorrhagic events [40]. Traditionally, it is categorized as either aPL negative or aPL positive, with approximately 41% of patients falling into the latter category in one case series [40,41]. Those who test positive for aPL may exhibit a clinical trajectory akin to primary APS patients [42]. However, understanding the precise role of aPL in this syndrome, its progression, and optimal treatment is severely hampered by the limited availability of reports. Additionally, less firmly established associations between cerebrovascular disorders and APS include reversible cerebral vasoconstriction syndrome [43] and Moyamoya arteriopathy [44].

Risk factors for each clinical phenotype of neurological involvement in APS have been suggested but are not yet fully understood. In a recent study by Volkov et al. [45], which assessed the presence of 20 different antiphospholipid antibodies (aPLs) and their correlation to various manifestations in APS, central nervous system (CNS) manifestations were found to be associated with a specific aPL profile. This profile included simultaneous positivity for IgG antibodies against prothrombin, phosphatidylglycerol, phosphatidylinositol, and annexin-5. Additionally, previous studies have suggested a correlation between cognitive deficits and higher titers of aPLs [46]. Female patients exhibited a higher prevalence of migraine, while epilepsy was more common in men [47]. Furthermore, chorea was found to be more frequent in young female patients with APS who carried aB2GPI [48,49].

A questioned issue, in particular from the neurological point of view, is the association between positive aPL and multiple sclerosis (MS) [50,51]. Neuropathologically, MS lesions are characterized by perivenular infiltration of myelin basic protein-activated CD4 T lymphocytes as well as reactive macrophages which orchestrate the massive inflammatory cascade within the CNS [52]. High frequencies of aPL antibodies are seen in autoimmune disorders other than systemic lupus erythematosus (SLE), not necessarily associated with thrombosis, such as in the immune thrombocytopenic purpura (ITP) [53] and in MS. The reported frequencies of positive aPL antibodies in MS ranges from 10% [54,55] to 44% [56] and to 88% [51,57,58]. Different methodologies and criteria of patient selection are the most likely causes of these discrepancies. The consequence is the lack of demonstration of a clear association between aPL antibodies and the clinical state or radiologic imaging data in MS patients. Bidot et al. [59] addressed this issue in 24 patients with relapsing–remitting MC, finding that during relapses, up to 80% of MS subjects had elevated titers of IgM aPL antibodies, but less than 50% had the same pattern on remission.

The attempts to demonstrate a specific antibody profile for the main neurological manifestations were largely unsuccessful. Volkov et al. [45] assessed the presence of 20 different aPLs in APS and tried to correlate each of them with a different manifestation, finding a correlation between a specific aPL profile and CNS manifestations. Previous studies have also suggested a correlation between cognitive deficit and higher titers of aPLs [46]. Female patients had a higher prevalence of migraine, while epilepsy was more common in men [47]. Chorea was more frequent in young female patients with APS carrying aB2GPI [48,49]. The high prevalence of APS and aPL positivity alongside other autoimmune conditions, notably SLE, has impeded the delineation of a distinct cognitive profile and the assessment of neuropsychiatric abnormalities in APS patients [60].

## 4. Neurovascular Issues

### 4.1. Pathological Findings

The positivity for aPLs increases the risk of a first ischemic stroke by 2-fold in young to middle-aged adults [61,62]; they then are routinely tested for in-clinical settings in the evaluation of unexplained stroke, and stroke prevention therapies may be beneficial even in the presence of asymptomatic carriers of aPLs [63]. APS is usually regarded as an autoimmune disease occurring in young patients, but data suggest an increasing prevalence of aPLs with aging. Indeed, a large study found that a third of people ≥80 years of age had aPLs [61]. Nevertheless, the relationship of aPLs to cerebrovascular disease in the elderly is less clear. In addition, few studies have tried to assess the relationship between APS and pathological evidence of stroke. This information, although limited, is crucial to evaluate the correlation of neuroimaging findings. A relatively recent study [64] addressed this issue, investigating the association of APS with pathological brain infarcts and cognitive and motor decline in aging in the Antiphospholipid Antibodies, Brain Infarcts, and Cognitive and Motor Decline in Aging (ABICMA) study [64]. This study enrolled ≥600 older, community-dwelling people who were followed up longitudinally with clinical, laboratory, and pathological data. In the two cohorts, the autopsy rate was ≥ 80%. A first interesting finding is that, among 607 subjects with neuropathological and aPL antibody data available, 142 subjects (23%) were positive for the overall aPL assessment at baseline. Most subjects had only one positive measure (*n* = 77), followed by two positive measures (*n* = 38) and then three measures (*n* = 23), and few (*n* = 4) had ≥4 positive measures. The cohort was old (mean age at death, 89 years; SD = 6.4), and then, vascular risk factors and vascular diseases were common. On neuropathology, half of the subjects (296 of 607, 49%) had a chronic infarct of any type (gross or microscopic, any location). A total of 118 subjects (19%) had gross infarcts without microinfarcts, 74 (12%) had microinfarcts without gross infarcts, and 104 (17%) both gross infarcts and microinfarcts. Subjects with and without aPL positivity had similar demographic and clinical features. In the primary logistic regression model adjusted for age and sex, the authors did not find that overall aPLs positivity at baseline was related to the presence of brain infarcts among older people. In addition, the small odds ratio (OR) suggests that the effect of aPLs on brain infarcts, even if present, is small. Moreover, a separate analysis for the size and location of infarcts did not show significant association.

In addition to human studies, animal models (mice) have provided some information about neuropathological findings in APS. In fact, the typical histopathological pattern of cortical tissue from APS mice was the microthrombotic occlusion of capillaries associated with mild inflammation [65]. The intravascular thrombosis was common in all vessels of any size. In a recent clinic–pathological study [66], aPL status was not associated with pathologically confirmed brain infarcts or cerebral atherosclerosis or arteriolosclerosis (all *p* ≥ 0.447) in a longitudinal older cohort.

In the series published by Zou et al. [67] on APS patients with neurological manifestations, histopathological data were available from the skin and brain in 16 and 3 patients, respectively. Skin biopsies showed inflammatory cells around the walls of the small vessels in superficial dermis, without thrombosis. Brain samples had focal softening, interstitial blood, and focal hemorrhage in the brain tissue. Both skin and brain biopsies suggested that APS-related cerebrovascular disease was mainly due to small-vessel involvement, with inflammatory cell infiltration, erythrocyte accumulation, neuron degeneration, and local demyelization. A previous study showed that microvascular involvement may be the most common pathological finding in patients affected by the catastrophic APS [68]. Microvascular thrombosis in APS may be a potential cause of multiorgan failure including, but not limited to, the lungs, brain, and kidneys [69].

One of the most reported associations of APS is with SLE, and both diseases frequently involve the central nervous system [70,71,72,73]. An autopsy study of neuropsychiatric SLE showed several types of brain lesions including global ischemic changes, parenchymal edema, microhemorrhages, glial hyperplasia, diffuse neuronal/axonal loss, resolved infarction, microthromboemboli, blood vessel remodeling, acute infarction, acute macrohemorrhages, and resolved intracranial hemorrhages [74]. In general, autopsy findings in neuropsychiatric SLE suggest that damage to the CNS is associated with cerebrovascular lesions [75]. The most commonly reported pathological findings include cerebral microvascular ischemia and thrombosis, noninflammatory lesions in small vessels, focal vascular occlusion, and microhemorrhage. Thrombosis of both large and small intracranial vessels, attributed in part to leukocyte coagulation and accelerated atherosclerosis, is also implicated in the pathogenesis of neuropsychiatric SLE. Additionally, immune complex deposition, complement activation, and vascular injury mediated by multiple autoantibodies play significant roles [76,77]. Beyond cerebrovascular involvement, brain histology in SLE patients reveals cerebral edema, vascular remodeling, wall calcification, neuronal and myelinated axonal loss, microinfarcts, diffuse ischemic changes, microglial proliferation, and reactive astrocytosis. These findings suggest that microglial activation may contribute to disease progression by impacting neuronal and synaptic structure and function. The aforementioned pathological alterations ultimately lead to focal or diffuse brain edema and diffuse endothelial injury, further disrupting the BBB [74].

### 4.2. Neuroradiological Patterns

Few studies have directly addressed the neuroimaging pattern of APS, although neurological manifestations are common. In fact, most studies are dated and do not present a detailed description of the neuroimaging patterns. In addition, the few available descriptions rely on small case series and do not refer to a common and standardized terminology, for example, in the description of markers of small-vessel disease (SVD). Thus, it appears difficult to bring these descriptions back to current standards of execution and neuroimaging reporting and, consequently, to extract highly sensitive and specific information. The most systematic approaches from the neuroradiological point of view were published in 1996 and 1998 [28,34]. However, care must be taken when using only neuroimaging findings to support a diagnosis of APS, as the latter are nonspecific for each individual disease.

#### 4.2.1. Small-Vessel Disease

Unfortunately, SVD involvement, mainly as an MRI marker of SVD, has not been addressed in the APS literature in a systematic way, and neuroradiological findings have not been described using a common terminology, as for example, STRIVE 1.0 and 2.0 standards [78].

In addition, the so-called MS-like manifestations in APS have a consistent overlap with SVD involvement, so in many cases, the cerebrovascular side of the disease is predominant [79]. In both conditions, multifocal MRI white-matter lesions are the predominant CNS manifestation [80,81]. In individuals with APS, small strokes may occur in the white matter of the brain and spinal cord, leading to lesions reminiscent of MS demyelinating plaques. However, these lesions are predominantly localized in the subcortical region, and recent research has identified multiple subcortical and cortical infarcts with demyelination across both brain hemispheres as characteristic MRI findings in APS patients. White-matter lesions, predominantly found in the periventricular area of the brain, were observed in nearly all cases studied [67]. In general, lesions associated with APS on MRI tend to be smaller in size, often localized in the subcortical region, and exhibit stability over time. Additionally, they may show improvement with anticoagulation therapy [82,83]. Normal cell counts and the absence of oligoclonal bands in cerebrospinal fluid (CSF) analysis also point toward APS [81]. Some researchers have proposed that aPLs might disrupt the integrity of the BBB, potentially facilitating immune cell access to the CNS compartment [59,84].

A notable correlation between cognitive dysfunction and MRI lesions has been observed in primary APS patients, even in those without CNS involvement [18]. Cognitive impairment affects 19–40% of aPL-positive patients and 42–80% of those with primary APS [18,46,60,85,86]. In a study involving 143 APS patients with moderate to high aPL titers, a linear correlation between aPL titers and cognitive dysfunction was observed [46]. The typical cognitive profile in primary APS often manifests as a subcortical pattern of mild cognitive impairment [85]. Two recent systematic literature reviews have indicated that the prevalence of cognitive impairment among individuals carrying aPL, primary APS, and secondary APS, collectively, falls within the range of 15% to 42% [87,88]. In a recent analysis of the APS ACTION registry, which aimed to delineate the baseline characteristics of approximately 800 patients with aPL positivity, cognitive impairment was observed in 85 (11%) patients [89]. Among patients with aPL positivity but without APS, 11 (7%) individuals exhibited cognitive impairment, whereas among those with both aPL positivity and APS, 74 (12%) had cognitive impairment. Additionally, the prevalence of cognitive impairment among APS patients was higher in those with thrombotic APS compared to those with obstetric APS, with figures of 53 (12%) versus 3 (4%), respectively. When considering the prevalence of cognitive impairment based on antibody type and the number of positive aPLs, patients with double and triple positivity showed a higher prevalence compared to those with single positivity, at 20 (12%) and 33 (12%) versus 17 (8%), respectively. Moreover, patients with single positivity for LAC displayed a slightly higher prevalence of cognitive impairment compared to those with single positivity for non-LAC antibodies, with figures of 14 (8%) versus 3 (6%), respectively. Multi-infarct dementia, classified as dementia, was detected in 2.5% of APS patients in the Euro-APS cohort, encompassing both primary and secondary APS cases [17]. Therefore, APS should be considered in young individuals with unexplained dementia [90]. Chronic ischemic cerebrovascular disease associated with aCL antibodies may underlie a vascular/multi-infarct dementia that could show partial improvement with APS therapy [91,92]. While reports suggest favorable cognitive outcomes with immunosuppression (e.g., rituximab) [92], the lack of robust evidence prevents the establishment of therapeutic guidelines for cognitive dysfunction in APS. Nevertheless, vascular damage may not be the sole pathophysiological mechanism. Reports have indicated findings consistent with degenerative dementia rather than multi-infarct dementia in elderly individuals positive for aPL [93]. Other studies, along with a meta-analysis, have emphasized a strong association with aCL antibodies [94]. Moreover, certain animal models have demonstrated that cognitive dysfunction can be induced by the intraventricular injection of neuronal-binding antibodies from APS patients [95], although others have failed to show an association with ischemic lesions [96]. Such findings support the notion of a direct impact of aPL on cognition. Additionally, aPL-mediated dysregulation of the dopaminergic system has been suggested [97]. Given the clinical similarities, consideration of an MS-like disease should also be included in the list of potential differential diagnoses.

**Figure 2:**
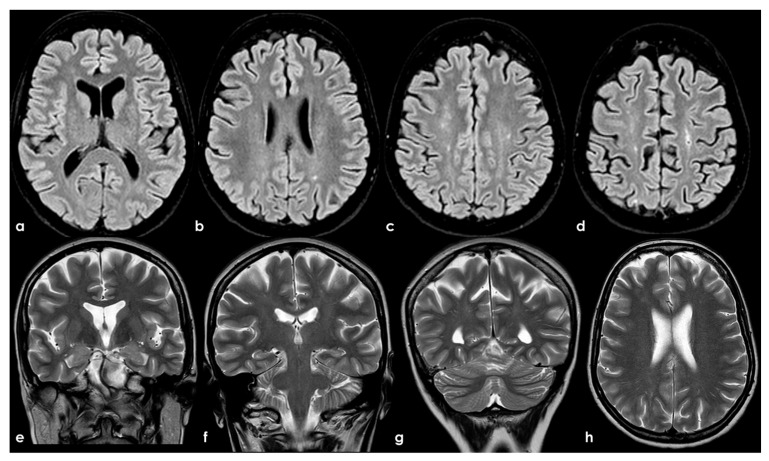
Brain MRI (axial FLAIR in panels (**a**–**d**), coronal T2W in panels (**a**–**g**), and axial T2W in panel (**h**)) showing small punctate white-matter hyperintensities in the centrum semiovale, with a trend to watershed distribution (panel (**d**)), and a mild increase in enlarged perivascular spaces in the subcortical white matter (panels (**e**–**h**)).

To date, as summarized by Hassan F et al. [98], most studies investigating cognitive impairment in individuals carrying aPL and in those with APS have been limited by small sample sizes and significant variations in cognitive-impairment detection methods, the specific aspects of cognition assessed, and the types of antibodies examined (e.g., aCL, LA, or aβ2GPI), as well as the laboratory cutoffs used to define positivity [99]. In general, aPL carriers represent a highly heterogeneous group with substantial variability in the prognosis and risk of cognitive impairment. The absence of standardized methods for quantifying aPL, which may also change over time, and fluctuations in cutoff levels for positivity, pose challenges in comparing findings across different studies. APS can be secondary to autoimmune diseases, which can independently affect the CNS and contribute to cognitive impairment. Moreover, aPL antibodies are more commonly detected in the elderly population, among whom cognitive impairment and dementia are prevalent [100]. Consequently, the precise frequency and mechanisms of cognitive impairment in APS, their correlation with aPL activity, and the optimal approaches to diagnosis and treatment remain uncertain [60]. In fact, in a recent systematic review [87] aiming to investigate the association between APS and cognitive dysfunction, the authors concluded that studies including neuroimaging biomarkers in APS/aPL-positive patients with cognitive dysfunction were scarce and heterogeneous; thus, multicenter studies with standardized image acquisition and international APS clinical and laboratory criteria are required. In Figure 2, an example of mild SVD involvement in a patient with APS (triple positivity) is proposed.

Estevez et al. [101] presented a series of extra-criteria aPL in 65 patients with SVD brain lesions, presenting MRI and clinical findings compatible with neurological APS but negative for clinical and laboratory APS classification criteria. The inclusion criteria were the presence of SVBL compatible with APS by MRI with six or more supratentorial subcortical T2 lesions, a Fazekas score ≥ 2 [102], and age ≥ 17 years. Exclusion criteria were the presence of any disease responsible for SVD or cardiovascular risk factors, positivity for aPL included in the classification criteria, over 70 years old, and being on immunosuppressive treatment. The rate of persistent extra-criteria aPL was 27.7%.

#### 4.2.2. Large-Vessel Involvement

A large series on the arteriographic features of 23 APS patients was published more than 20 years ago [28]. Seventeen patients (74%; 12 women, average age 40 years) exhibited abnormal catheter angiograms. Among these, ten patients (43%) displayed solely intracranial abnormalities, of which nine were arterial and one was venous (dural sinus thrombosis). Six patients (26%) manifested solely extracranial arterial abnormalities, and one patient (4%) had both intracranial and extracranial arterial abnormalities. Thirteen patients exhibited infarctions on CT or MR examinations, all of which were arterial events. Among the remaining four patients, one displayed dural sinus thrombosis on MR images, while the other three had normal CT or MR imaging studies despite a clinical course compatible with transient ischemic attack. Notably, the one instance of dural sinus thrombosis occurred in the right transverse sinus without associated infarction. Two major types of intracranial arterial abnormalities were observed: stem occlusions of major arteries or branch occlusions, typically solitary, and multifocal sites of arterial narrowing and widening suggestive of (but not proven to be) vasculitis. Among the patients, six displayed intracranial arterial occlusions, occurring in the basilar (BA) (two patients), middle cerebral (MCA) (two patients), and anterior cerebral (ACA) (one patient) arteries, as well as in MCA branches (one patient). Additionally, four patients exhibited multiple sites of arterial narrowing and dilatation, suggestive of vasculitis. Of these, one patient showed a clinical course clearly indicative of CNS vasculitis and improved clinically with long-term immunosuppressive therapy. However, in the remaining three patients, the long-term clinical course did not conclusively suggest CNS vasculitis as the likely cause of abnormalities seen at arteriography. Instead, a noninflammatory vasculopathy was considered the probable cause. Extracranial arterial abnormalities, observed in seven patients (32%), were classified into three types: common carotid or internal carotid artery (ICA) stenosis or occlusion (two patients), stenoses or occlusions of the origin of two or more great vessels (Takayasu-like pattern, four patients), and narrowing of the ICA in a pattern typical of atheromatous disease (one patient, age 53 years). Notably, one patient with extracranial carotid artery occlusion was found to have aortic thrombus, presumed to be the source of an embolus to the ICA, and underwent successful ICA thrombectomy. In this angiographic series, patients ≥ 65 years old were excluded, mainly to minimize the potential impact of traditional vascular risk factors and atherosclerosis as a cause of stroke. The most important finding was that arterial thrombosis is more frequent than venous thrombosis in APS patients, in line with previous and following studies. One of the most interesting finding is the “vasculitis-like” pattern shown in one patient. It is not surprising the lack of confirmation of the vasculitis hypothesis on leptomeningeal and brain biopsy, read with today’s knowledge about the subtyping of primary CNS angiitis according to the size of involved vessels, as outlined in the recent guidelines [103]. In fact, large- and medium-vessel involvement is usually associated with a negative biopsy because a biopsy is able to sample only small vessels. However, it is possible that these angiographic findings might indicate an underlying noninflammatory vasculopathy, rather than true vasculitis. Old autoptic series did not show vasculitis in APS patients [104]. In fact, most cases of large-vessel occlusive disease in APSA patients have been found to be thrombotic in nature, often associated with underlying mural abnormalities [105]. While a few cases have shown features of vasculitis with histologic evidence of a transmural lymphocytic infiltrate, such occurrences are rare and usually associated with an independent underlying disease [105]. Interestingly, abnormalities such as concentric intimal hyperplasia, fibrous occlusions, and thrombi—without evidence of vasculitis—have been observed in small leptomeningeal arteries in APS patients with ischemic cerebrovascular events [106]. It is plausible that similar mechanisms affecting intracranial arteries could explain the arteriographic “vasculitis-like” findings.

**Figure 3:**
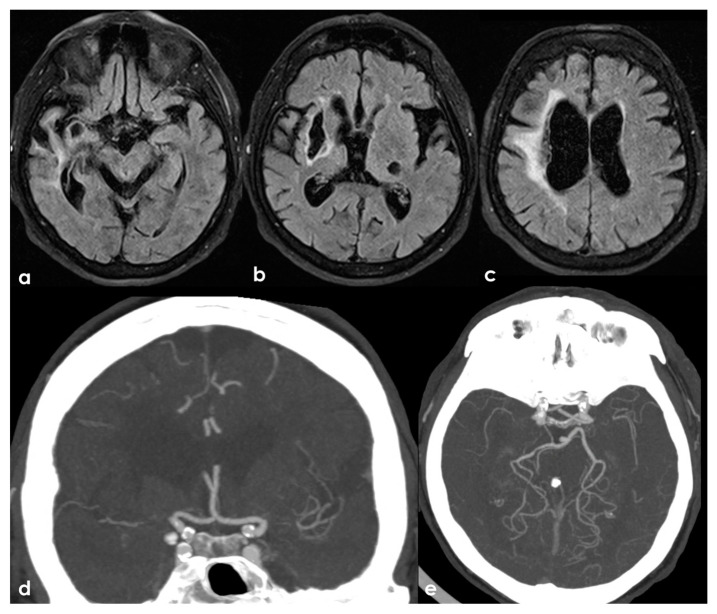
A remote ischemic lesion in the right MCA territory is illustrated in the axial FLAIR sequence of the brain MRI (panel (**a**–**c**)) with the corresponding vascular imaging on a CT angiography with minimum intensity projection/multiplanar reconstruction (MIP/MPR) (panel (**d**,**e**)) in the coronal and axial plane, respectively. M1 MCA on both sides is occluded with a tiny network of small vessels partially contributing to supply M2 MCA.

**Figure 4:**
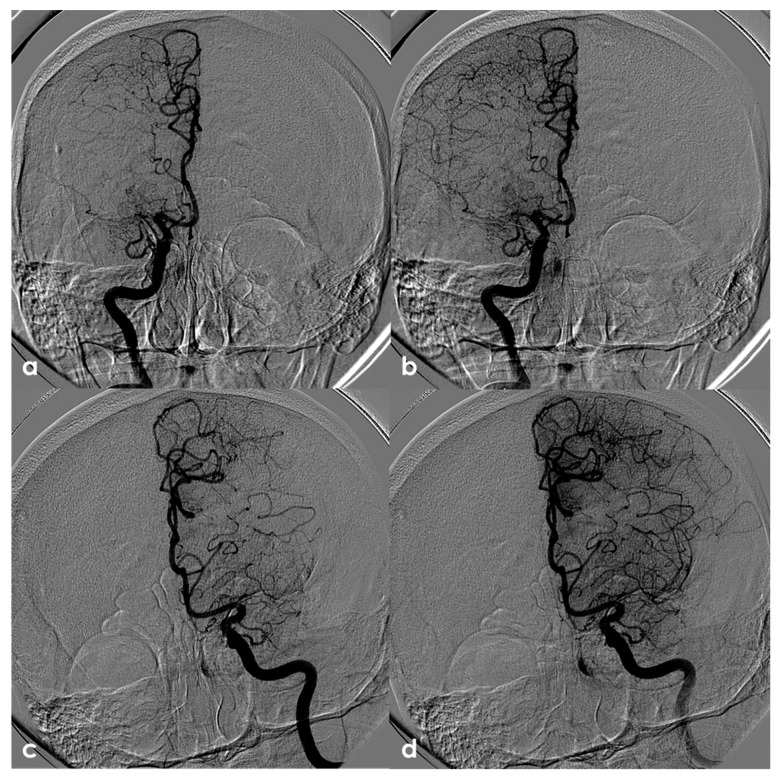
Digital subtraction angiography (DSA) of the same patient as in Figure 3 from a (**right**) (panels (**a**,**b**)) and (**left**) (panels (**c**,**d**)) ICA injection in PA view. Panels (**a**,**c**) are an early arterial phase, and panels (**a**,**b**) are a mid– late arterial phase. On both sides, the M1 MCA after its origin appears steno-occluded and is substituted by a network of collateral vessels involving the perforating arteries.

One of the most conflicting and discussed associations is between APS and large-vessel arteriopathy, in particular moyamoya arteriopathy (MMA). The detailed description of MMA is outside the aims of this review. The several conditions contributing to MMA, from primary MMA to the secondary causes, share the neuroradiological pattern of unilateral or bilateral intracranial distal internal carotid artery stenosis or occlusion with the subsequent development of prominent leptomeningeal, parenchymal, and dural anastomotic channels. The diagnostic criteria were recently updated [107], and multidisciplinary guidelines were published [108,109]. Sneddon’s syndrome was first described in 1965 by the British dermatologist I.B. Sneddon, who noted the association of a noninflammatory obliterans arteriopathy that affected the dermis (livedo reticularis) and the central nervous system (both ectodermal derivatives). From the neurological point of view, Sneddon’s syndrome has been documented to produce clinical features of acute transient or permanent cerebral ischemia and progressive cognitive impairment up to vascular dementia. The association of Sneddon’s syndrome with APS and aPL was postulated early [110,111]. In a literature review published in 2014, Zhang et al. [44] found 16 well-described cases of MMA with aPL, all presenting with the onset of cerebral ischemia. They described a further case, and 17 patients were included in the review (8 males and 9 females with a mean age of 16 ± 14 years, range of 1–43 years). In total, 11/17 (65%) of the cases were accompanied by other comorbidities (4 had Down’s syndrome, 3 had autoimmune thyroid disease, 3 were post-infective arteriopathy, 1 had Sneddon’s syndrome, 1 had SLE, 1 had type 1 diabetes mellitus, 1 had Noonan syndrome, 1 had adenomyosis, and 3 had positive autoantibodies without explicit autoimmune disease). Only 5/17 (29%) of cases fulfilled the 2006 criteria of definite APS. The remaining 12/17 (71%) MMA cases had coexisting aPL that did not fulfil the APS criteria. The connection between MMA and aPL remains murky. It has been suggested that aPL formation might be linked to damaged vascular structures, thrombosis due to abnormal vasculature or altered blood flow, or an unidentified systemic condition underlying MMS. Additionally, aPL may exacerbate thrombosis and recurrent ischemic events [112]. When aPL appears in MMA, other underlying conditions should be considered. Figure 3 shows an example of bilateral MCA occlusion in a patient with APS secondary to SLE with a neuroimaging evolution coherent with an MMA-like development of collateral circulation (Figure 4).

It is intriguing that aPLs can activate endothelial cells and stimulate the proliferation of vascular cells in the intima and media, without forming intraluminal thrombi. This phenomenon, known as nonthrombotic proliferative vasculopathy (PV) associated with aPL (PV-aPL), can result in critical stenoses in pulmonary, visceral, and peripheral arteries. Unlike arterial thrombi seen in APS or atherosclerotic plaques, which typically cause abrupt, short-segmental stenosis or occlusion, aPLs can induce diffuse vascular wall proliferation, leading to long-segmental stenosis. PV-aPL is a rare cause of vascular stenosis and might represent an underrecognized form of extra-criteria manifestations of APS. However, there is limited understanding of the angiographic features of PV-aPL involving cerebral and cervical arteries. A series of 11 APS patients was recently published [113], using MRA to analyze the angiographic characteristics of PV-aPL affecting cerebral and cervical arteries in patients with aPLs who presented neurological symptoms. The described cohort included six men and five women (median age of 42 years, range: 34–61), exhibiting diffuse luminal narrowing affecting the cerebral and cervical arteries. The clinical presentation was variable: six presented with acute neurological symptoms like hemiplegia, syncope, diplopia, or memory impairment, while the remaining five complained of chronic headaches lasting from 1 month to 5 years. About the aPL profile, six had triple-positive aPL profiles, four had double-positive profiles, and one had a single-positive profile. Two patients had coexisting autoimmune conditions, including one with SLE and one with Graves’ disease. All 11 patients exhibited diffuse luminal narrowing in major extracranial and cerebral arteries: 5 (45.5%) in the ICA, 2 (18.2%) in the MCA, 2 (18.2%) in the vertebral artery (VA), 1 (9.1%) in the BA, and 1 (9.1%) in the posterior cerebral artery (PCA). One patient had long-segmental stenosis in the entire left ICA extending into the left MCA, while three patients exhibited relatively short-segmental narrowing. Additional abnormalities observed in the same vascular territory included focal stenosis in eight patients (72.7%), distal occlusion in three (27.3%), and an aneurysm in one (9.1%). Six patients underwent high-resolution vascular wall MRI (VW-MRI) to assess vascular wall and intraluminal changes. VW-MRI revealed concentric thickening of the vascular walls of the ICA and/or MCA in four patients and mild eccentric thickening of the vascular walls of the ICA or BA in two patients. Contrast enhancement of the vascular walls was observed in three patients, including one with concentric changes and three with eccentric changes. None of these six patients exhibited intraluminal occlusion or thrombus or intramural hematoma. A remarkable angiographic finding in PV-aPL is the extensive long-segmental stenosis of medium-sized extra- and intracranial arteries, including the carotid, basilar, and proximal cerebral arteries. A similar angiographic pattern observed in the aorta and its main branches in a patient with APS has been likened to Takayasu arteritis-like noninflammatory vasculopathy. However, none of the patients in our study met the criteria for primary systemic vasculitis, including Takayasu arteritis or giant cell arteritis, whereas Ree et al. [114] reported a 6.3% prevalence of APS in primary systemic vasculitis. Many patients with PV-aPL exhibited short-segmental (focal), abrupt stenosis, and distal occlusions suggestive of atheromatous or thrombotic lesions [115]. Patients with PV-aPL may be at a higher risk of atherosclerosis, as indicated by a 2.5-fold higher risk of developing atherosclerotic plaques in carotid and femoral arteries compared to healthy controls [116,117,118]. The association between atherosclerosis and PV development remains unclear. Some studies suggest that aPLs might drive both PV and atherosclerosis. Previous studies [118] have reported two major types of intracranial arterial abnormalities associated with aPLs: stem occlusions of major arteries or branches and multifocal sites of arterial narrowing and widening. However, as previously signaled, several case studies have described moyamoya-like vasculopathy in patients with aPLs [44,119]. The long-segmental stenosis observed in these patients may belong to the latter category, but luminal angiography methods such as MRA have limited ability to visualize underlying pathological processes in these blood vessels. VW-MRI can directly image pathological changes in vessel walls. Concentric wall thickening and enhancement observed on VW-MRI suggest vasculitis [120], while intraluminal thrombosis is improbable, but it shows intraluminal enhancement. This study strongly indicates that the characteristic long-segmental stenosis observed in these patients is unlikely attributable to diffuse intraluminal thrombus formation, but potentially is due to PV. The lack of an intraluminal thrombus, concentric vascular wall thickening on VW-MRI, normal D-dimer levels, and disease progression under antithrombotic drugs strongly suggests that long-segmental stenosis was not induced by classical APS with intraluminal thrombus. [121,122]. Additionally, normal CRP levels and a lack of response to corticosteroids make inflammatory vasculopathy less likely, although some patients exhibited mural contrast enhancement.

Proliferation of the vascular wall may be driven by aPLs, which can activate endothelial cells releasing proliferative cytokines [106]. These cytokines promote the proliferation of cells in the intimal and medial layers, leading to concentric stenosis similar to transplant vasculopathy and pulmonary arterial hypertension. It is unclear whether aPL titers and/or certain aPL profiles are associated with the extent and progression of PV-aPL. In a previous study by Djokovic et al. [123], the presence of aB2GPI IgG might be associated with more serious cerebrovascular events. A reduction in aPLs through plasmapheresis or the depletion of B cells or plasma cells might improve long-term prognosis. Further research is needed to define the optimal treatment for PV-aPL, as there is no official management guideline. Notably, PV-aPL should not be confused with the cerebral proliferative angiopathy described by Lajaunias [124,125].

#### 4.2.3. Brain Parenchyma

The link between aPL and ischemic stroke has been established for some time. However, there remains a dearth of information regarding the neuroimaging pattern of aPL-related stroke (aPL-stroke). The limited studies on this topic are marred by various constraints, such as the selective nature of neuroimaging evaluations, failure to account for alternative stroke causes beyond aPL, or drawing conclusions from heterogeneous disease groups including cerebral venous thrombosis, seizure, or migraine, alongside cerebral infarction [22,28,34,126]. Moreover, these studies predominantly stem from the 1990s and thus fail to reflect the significant diagnostic and management advancements in stroke and vascular risk factors.

**Figure 5:**
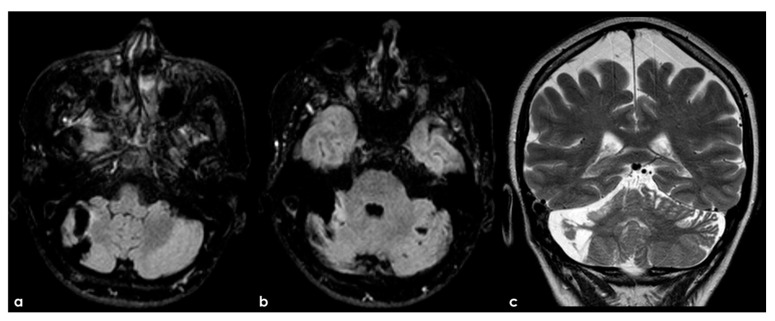
An example of multifocal cerebellar ischemic lesions in a patient with APS (double positivity). Brain MRI (axial FLAIR in (**a**,**b**), coronal T2W sequence in (**c**) shows the poromalacic evolution of multiple ischemic lesions involving both cerebellar hemispheres (right ≥ left). No causes other than APS were identified in this patients.

From a neurologist’s perspective, understanding the distinct characteristics of aPL-stroke holds potential in mitigating cryptogenic stroke cases, in particular in comparison with occult atrial fibrillation (AF) -associated cryptogenic stroke. In this subset of patients with cryptogenic stroke, a notably high aPL-positivity rate was reported across all age groups [127]. Distinguishing features of aPL-stroke from AF-related stroke (AF-stroke) could streamline aPL screening in such cases, thereby reducing diagnostic ambiguity. Furthermore, recognizing aPL-stroke could aid in preventing the inappropriate use of direct oral anticoagulants (DOACs) in undiagnosed aPL-stroke patients, given recent reports highlighting DOAC-related risks in APS [128,129,130]. In a recently published series [131] on 129 patients with acute ischemic stroke and positive aPL compared with 333 patients with AF-related acute ischemic stroke, some interesting information has been provided. First, only 56/129 (45.7%) of patients in the aPL group had APS as a cause of stroke; large artery atherosclerosis being the most prominent cause. In the whole cohort, the median [interquartile range] age was 75 [65,66,67,68,69,70,71,72,73,74,75,76,77,78,79] years, and 216 (55.5%) were males. aPL-stroke patients were significantly younger than AF-stroke patients, but they were more likely to be smokers and less likely to have a stroke history and use antithrombotics before the index stroke. In general, the stroke severity was milder in the aPL-stroke group. In the aPL-stroke group, a higher proportion of patients exhibited a single small lesion, whereas over half of the AF-stroke patients displayed a large territorial infarction. In aPL-stroke patients, the overall diffusion-weighted imaging (DWI) lesion volume was notably smaller compared to AF-stroke patients. Additionally, a significant majority (over 80%) of aPL-stroke patients exhibited no relevant artery occlusion, while more than half of AF-stroke patients experienced occlusion of the pertinent artery. The proportion of multi-territory lesions was comparable between the two groups (aPL-stroke: 16 [28.6%]; AF-stroke: 76 [22.8%]; *p* = 0.44). However, upon analyzing only patients with multi-territory lesions, aPL-stroke patients tended to have small (≤15 mm) scattered lesions, whereas AF-stroke patients predominantly displayed confluent (≥15 mm) lesions with additional lesions. Furthermore, the total DWI lesion volume was smaller in aPL-stroke patients compared to AF-stroke patients with multi-territory lesions. Consequently, in the multivariate analyses, the largest lesion size ≤ 15 mm in diameter, smaller total DWI lesion volume, and the absence of relevant artery occlusion were independently associated with aPL-stroke with an odds ratio of 5.07 (2.37–10.85), 1.28 (1.12–1.45), and 6.93 (2.78–17.27), respectively. Twenty-one patients within the aPL-stroke group were diagnosed with definite APS. Interestingly, the definite APS-stroke patients exhibited similar clinical, laboratory, and imaging characteristics compared to the broader aPL-stroke group. Upon comparing the definite APS- and AF-stroke groups, the results were generally consistent with the aforementioned analysis comparing the aPL- and AF-stroke groups. Moreover, the infarct burden among patients with multi-territory lesions was notably lower in the definite APS-stroke group. Some of these issues are not always respected in clinical practice, as shown in Figure 5.

**Figure 6:**
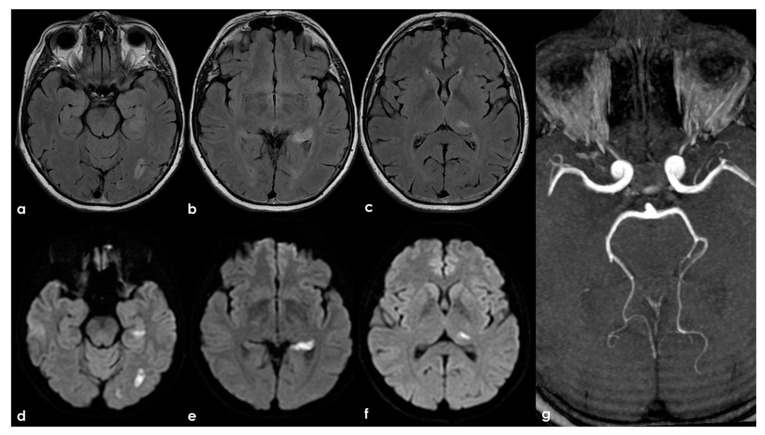
Brain MRI (axial FLAIR in panels (**a**–**c**) and the corresponding DWI slices in panels (**d**–**f**)) showing multifocal ischemic lesions in the left PCA territory with a patent PCA on MRA (panel (**g**)).

In addition, Figure 6 shows the presence of multiple ischemic lesions in the same arterial territory, and this issue has not been clearly addressed in the multiplicity of lesions described in the abovementioned paper [131].

**Figure 7:**
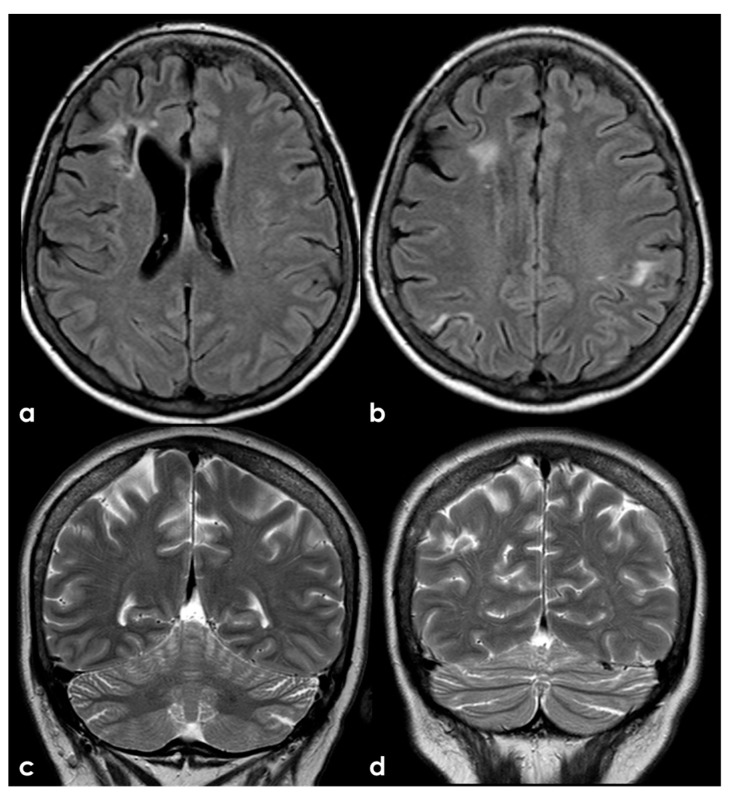
Brain MRI (axial FLAIR in panels (**a**,**b**) and coronal T2W sequence in panels (**c**,**d**)) showing multiple cortico-subcortical ischemic lesions on both hemispheres and only few mildest SVD markers.

A different situation is illustrated in Figure 7, where the multiple ischemic lesions are in different vascular territories.

**Figure 8:**
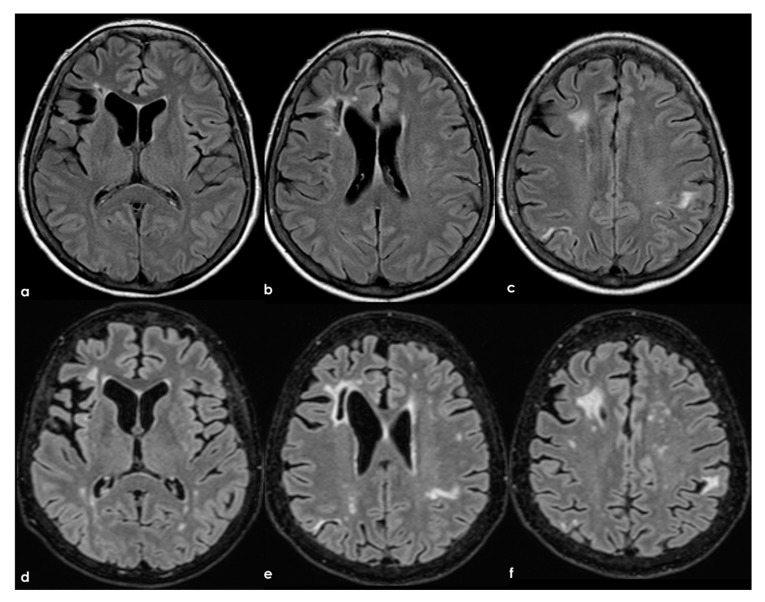
Brain MRI (axial FLAIR) at baseline (panels (**a**–**c**)) and after 8 years (panels (**d**-**f**)), showing the increase of WMHs in the subcortical white matter.

In the longitudinal evolution of neuroradiological patterns, SVD markers might appear, as in Figure 8 (same patient as in Figure 7 but 8 years later and without new clinical events on anticoagulant treatment).

However, the precise mechanism through which aPL triggers ischemic stroke has remained elusive. It has been proposed that the presence of aPL may exacerbate atherosclerosis and contribute to the development of cardiac issues, ultimately culminating in ischemic stroke [2,29,132]. In the most recent series [131], the proportion of patients exhibiting multi-territory lesions in aPL-stroke cases closely mirrored that of AF-associated cardioembolic stroke, accounting for nearly 30% of cases. When coupled with the milder neuroimaging characteristics observed in aPL-stroke, these findings suggest that positive aPL may lead to ischemic stroke via small-sized emboli originating proximally to large cervical vessels, rather than through direct involvement of intracranial vessels. These proximal emboli could originate from cardiac sources or thrombi formed at the walls of proximal arteries. While changes in cardiac valves in APS have been previously implicated in stroke [29], the prevalence of subclinical valve lesions was not as high in aPL-stroke compared to AF-stroke. Instead of originating from cardiac sources, the culprit thrombus in aPL-stroke cases may develop in the arteries proximal to large cervical vessels. Factors such as a higher proportion of smokers, elevated LDL levels, and a similar prevalence of hypertension and hyperlipidemia in aPL-stroke patients compared to AF-stroke patients, despite their younger age, may act as “second hits” that precipitate thrombus formation at sites of minor endothelial injury [133]. Thirty percent more of aPL-stroke cases manifested with a solitary minor lesion. Such a lesion configuration could stem from either embolism, characterized by minute thrombi at proximal sites, or thrombosis directly affecting intracranial arteries. A complex underlying pathophysiology warrants consideration contingent upon the lesion configuration in aPL-stroke. Investigating the mechanisms behind aPL-stroke concerning diverse lesion patterns would be invaluable in bolstering secondary prevention efforts.

#### 4.2.4. SLE with APS

There has been limited reporting of the differences in MR findings in patients with SLE with APS and those without APS [134]. A larger series (256 patients) was described by Kaichi et al. [135], aiming to characterize the spectrum of MR findings of SLE with and without APS. The most common finding was WMH (42%), followed by infarcts and infarct-like lesions (27%). Among this last subgroup, only 8 of the 69 (12%) harbored stenotic lesions on major intracranial arteries on MRA: in 6/23 (26%) patients with large territorial, 2/23 (9%) with lacunar, and 2/11 (18%) with borderzone infarcts. No stenotic lesion on the relevant artery was seen in patients with basal ganglia lesions and acute micro-embolism in the cortex and/or subcortical white matter. More patients with than without APS demonstrated abnormal findings (73% versus 53%). The incidence of large territorial, lacunar, localized cortical, and borderzone infarcts; acute microemboli; basal ganglia lesions; callosal lesions; and stenotic arterial lesions was higher in patients with APS than in those without APS. Large territorial, localized cortical, and borderzone infarctions; basal ganglia lesions; lacunar infarcts; stenotic arterial lesions; and the total number of patients with abnormal findings were significantly associated with APS. The incidence of large territorial infarctions in the cerebellum was significantly higher in patients with APS than in those without APS (*p* = 0.02). Localized cortical infarctions in the MCA area were significantly associated with APS (*p* = 0.01). Bilateral but not unilateral borderzone infarcts were associated with APS (*p* = 0.01). All basal ganglia lesions were in the anterior zone (7/7). Stenotic arterial lesions were seen in the ICA and MCA. WHM, lacunar infarcts, and the total number of patients with abnormal findings were significantly associated with age.

## 5. Extra-Criteria Neurological Patients

The past diagnostic criteria of APS are highly specific, but they are not very sensitive for APS diagnosis [12]. The updated criteria [cite] have introduced further clinical domains to overcome the problem of extra-criteria APS diagnosis [97,136], but neurological issues are still neglected. Moreover, a seronegative status for the three autoantibodies included in the criteria might not exclude APS, and several other autoantibodies have been suggested to be tested: IgA aCL or ab2GPI, Abs to annexin V, prothrombin (PT), phosphatidylserine/prothrombin (PS/PT), phospholipids other than cardiolipin such as phosphatidylethanolamine (PE), and domain I of b2GPI, etc. [137,138,139].

## 6. Conclusions

APS is a complex disease, and its neurological involvement appears multifaceted and not yet fully characterized. Some clinical and neuroradiological manifestations are not included in the diagnostic criteria, despite significant advancements in their recent update. In particular, the concept of arterial and venous thrombosis is not easily and unequivocally applicable to the description of cerebrovascular involvement in the disease, and several elements still fall outside the criteria. Specifically, the involvement of intracranial large vessels appears poorly characterized, and even the components of SVD are described incompletely and may account for many of the previously described inflammatory manifestations.

## Figures and Tables

**Table 1:** Definition of the high-risk and moderate-risk CVD profile for AT [14].

High CVD Risk Factors (Any of the Following at the Time of the Event)	Moderate CVD Risk Factors (≥3 of the Following at the Time of the Event)
Severe arterial hypertension ^a^	Arterial hypertension on treatment, or with persistent systolic BP ≥ 140 mm Hg or diastolic BP ≥ 90 mm Hg
Chronic kidney disease ^b^	Current tobacco smoking
Diabetes mellitus with organ damage or long disease duration ^c^	Diabetes mellitus with no organ damage and short disease duration ^e^
Severe hyperlipidemia ^d^	Moderate hyperlipidemia on treatment, Total cholesterol above the normal range but <310 mg/dL (8 mmol/L), or LDL cholesterol above the normal range and <190 mg/dL (4.9 mmol/L)
	Obesity ^f^

^a^ systolic blood pressure (BP) ≥ 180 mm Hg or diastolic BP ≥ 110 mm Hg; ^b^ estimated glomerular filtration rate ≤ 60 mL/min for more than 3 months; ^c^ type 1 for ≥20 years; type 2 for ≥10 years; ^d^ total cholesterol ≥ 310 mg/dL (8 mmol/L) or low-density lipoprotein (LDL)–cholesterol ≥ 190 mg/dL (4.9 mmol/L); ^e^ type 1 < 20 years; type 2 < 10 years; ^f^ BMI ≥ 30 kg/m^2^.
